# State Medicine

**Published:** 1875-02

**Authors:** 


State Medicine.—Every few years some one gets up a bill in the Legislature to "protect" competent medical practitioners by fining incompetent ones, and forcing them out of the field. This is to be accomplished by the exclusion of those who have no diploma, or by examining boards, to ascertain the qualifications of those who would practice medicine irrespective of diploma-holding. Something of the last-mentioned kind is now pending in the Legislature of Tennessee.

It is passing strange that those who favor such schemes will not remember that, in either case, they throw themselves into the hands of politicians, who cannot know anything of such matters, and who care less, if possible, than nothing. Suppose our present Legislature appoint such a Board from among us, or several Boards, as contemplated—at Nashville, Memphis, and Knoxville—say of about two dozen doctors in all—to say who, of as many thousand, should practice medicine, would not the members, upon returning home, find their constituents, like so many yellow-jackets, in high stinging mood? Do not *they* know who does them most good when sick, and who lucks best?

Qualifications! Bah! Pretty is that pretty does! This is the logic of the masses, and the king of our country is the aggregate of the masses. And what would the other one thousand nine hundred and seventy-six doctors think?

And of the diploma phase: One man already proposes, in our newspapers, that should the bill assume that shape, he will guarantee the diplomaless a full supply for a few dollars a piece!

And thus is an entire profession blackguarded because of the indiscretion of a few of its members.

This journal has set its face, like flint, for a quarter of a century, against the meddling of non-medical men with medical affairs, its motto being, "To medical men belong medical matters." Our State Society has taken similar ground. Every medical man we have seen and conversed with upon the subject is dead set against the pollution that law alone can bring to medicine. The language of each is, "If I cannot stand without props, let me fall,"; and this is the language of common sense.

Let the physicians of each county organize, and every three or six months publish, in the county paper, the names of the physicians of the county. Does not Congress alone judge who are Congressmen?—and are not physicians alone judges of who are physicians? Does not every Church do this? Did not the Massachusetts Legislature declare that a regular bred physician was a member of the Masschusetts Medical Society, and refuse to know anything else in regard to medicine?

Hew to the line, brethren, and let others get out of the way of the chips, and "shinny on their own side."—*Nashville Journal of Medicine and Surgery.*

